# Supplementary material for: Learning induces coordinated neuronal plasticity of metabolic demands and functional brain networks
Source: Commun Biol. 2022 May 9;5:428. doi: 10.1038/s42003-022-03362-4 (PMC9085889; doi:10.1038/s42003-022-03362-4)
Supplement: Supplementary file 2 — Supplementary Material [file 42003_2022_3362_MOESM2_ESM.pdf]

SUPPLEMENTARY MATERIAL for

Learning induces coordinated neuronal plasticity of metabolic demands and functional brain networks

Sebastian Klug[1#], Godber M Godbersen[1#], Lucas Rischka[1], Wolfgang Wadsak[2,3], Verena Pichler[2,4], Manfred Klöbl[1], Marcus Hacker[2], Rupert Lanzenberger[1], Andreas Hahn[1*]

[1] Department of Psychiatry and Psychotherapy, Medical University of Vienna, Austria

[2] Department of Biomedical Imaging and Image-guided Therapy, Division of Nuclear Medicine, Medical University of Vienna, Austria

[3] Center for Biomarker Research in Medicine (CBmed), Graz, Austria

[4] Department of Pharmaceutical Sciences, Division of Pharmaceutical Chemistry, University of Vienna, Austria

SUPPLEMENTARY FIGURES

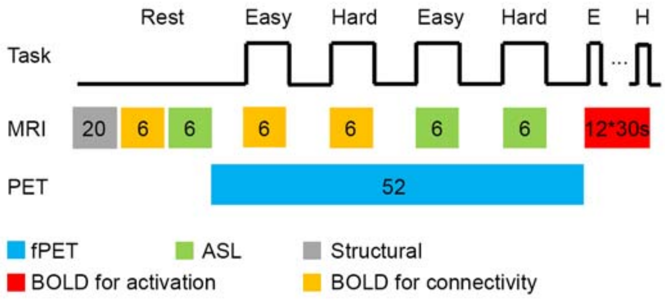

Supplementary figure S1: *PET/MRI measurement procedure*. Data acquisition included a T1-weighted structural scan (gray, 8 min) as well as BOLD imaging (orange, 6 min) and ASL (green, 6 min) at resting state. After that, [^{18}F]FDG fPET imaging started (blue, 52 min) with simultaneous recording of BOLD (orange) and ASL (green). During these MRI sequences, continuous task performance was carried out at two levels of difficulty (easy and hard in randomized order). BOLD sequences obtained at rest and during the task (orange) were used to estimate functional connectivity and subsequently for metabolic connectivity mapping. ASL and fPET were used to quantify indices of task-induced neuronal activation as CBF and CMRGlu, respectively. After fPET was completed, the last MRI acquisition again comprised BOLD imaging. Here, a conventional block design was employed as a third estimate of task-specific neuronal activation (red, 12 randomized blocks of easy, hard and control conditions, 30 s each with 10 s baseline in-between, total 8.17 min). Numbers indicate measurement time in minutes unless indicated otherwise. This figure was adapted from Hahn et al. (2020)[1] under the CC BY 4.0 license.

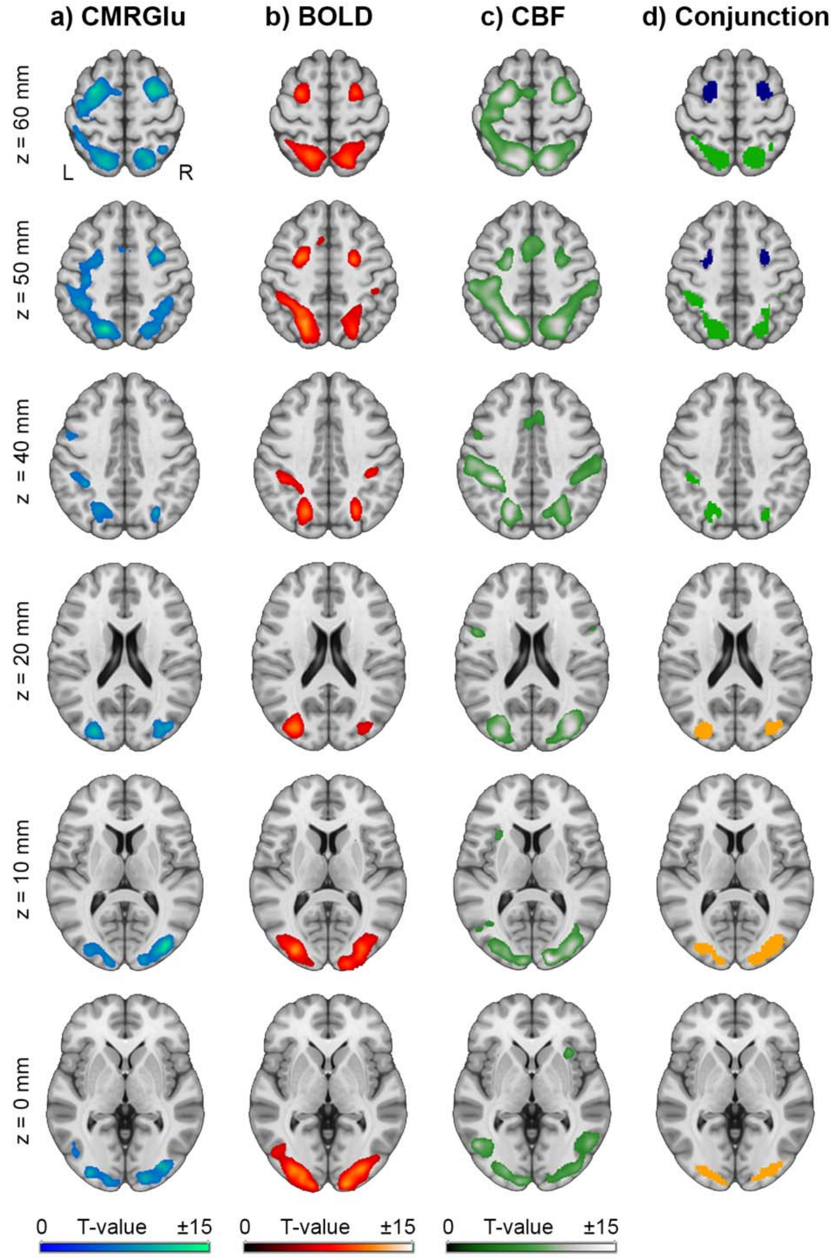

Supplementary figure S2: *Multimodal parameters of neuronal activation*. Task-induced changes as identified by imaging of the cerebral metabolic rate of glucose (CMRGlu, **a**), blood-oxygen level dependent signal (BOLD, **b**) and cerebral blood flow (CBF, **c**). Group maps were derived from the first PET/MRI measurement (n=39) and thresholded at $p<0.05$ FWE-corrected voxel level. Dice coefficients were 0.57 (CMRGlu vs BOLD), 0.51 (CMRGlu vs CBF) and 0.48 (CBF vs BOLD). The conjunction map (**d**) shows the spatial overlap in metabolic demands as computed by the intersection across the three imaging modalities (orange: occipital cortex,

green: intraparietal sulcus, blue: frontal eye field). Those regions showing an overlap in task-specific activation were used as target regions for the subsequent metabolic connectivity mapping (MCM) analysis. Axial slices are shown in neurological convention (left is left).

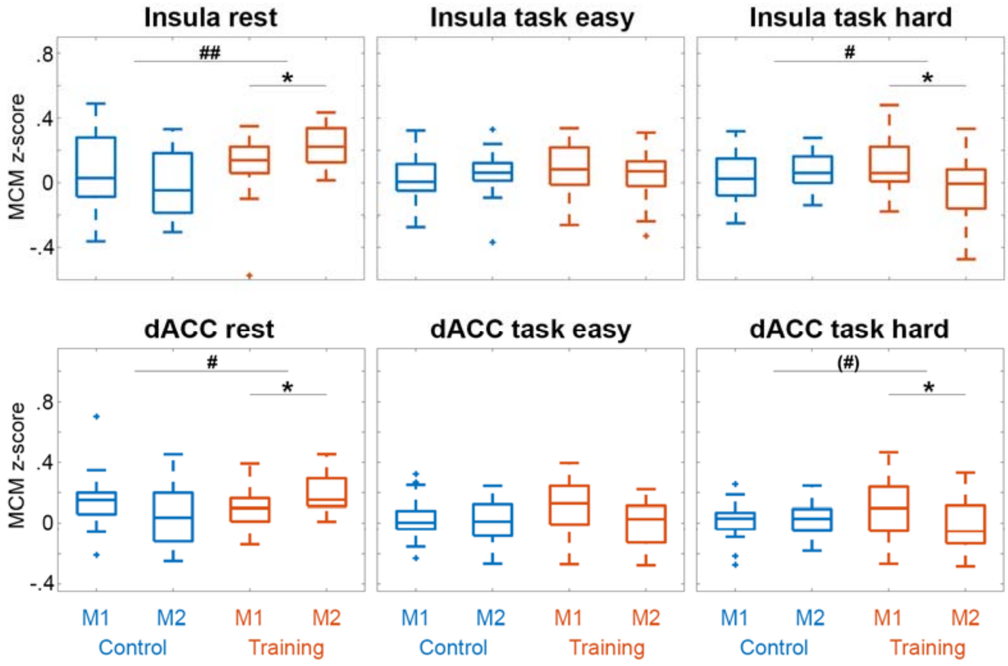

Supplementary figure S3: Learning-induced changes in MCM when defining the occipital cortex as target region from the intersection between task-specific CMRGlu and BOLD signal changes. Compared to figure 3 (i.e., the target region defined from CMRGlu, BOLD and CBF), these results were slightly weaker but still remained significant. Boxplots show the MCM z-scores of the insula and dorsal anterior cingulate cortex (dACC). Post-hoc comparisons indicate significant differences for the group*time interaction ($^{(\#)}$p=0.06, $^{\#}$p<0.05, $^{\#\#}$p<0.01) and for the differences between the two measurements (*p<0.05, **p<0.01), corrected for multiple comparisons with the Bonferroni-Holm procedure. M1 indicates the first PET/MRI measurement and M2 the second, with training group in red (n=21) and control group in blue (n=20). Boxplots indicate median values (center line), upper and lower quartiles (box limits) and 1.5*interquartile range (whiskers).

SUPPLEMENTARY REFERENCES

1. Hahn, A. *et al.* Reconfiguration of functional brain networks and metabolic cost converge during task performance. *eLife* **9**, e52443 (2020).
