# Supplementary material for: Learning induces coordinated neuronal plasticity of metabolic demands and functional brain networks
Source: Commun Biol. 2022 May 9;5:428. doi: 10.1038/s42003-022-03362-4 (PMC9085889; doi:10.1038/s42003-022-03362-4)
Supplement: Supplementary file 3 — Description of Additional Supplementary Files [file 42003_2022_3362_MOESM3_ESM.pdf]

DESCRIPTION of ADDITIONAL SUPPLEMENTARY FILES for

Learning induces coordinated neuronal plasticity of metabolic demands and functional brain networks

Sebastian Klug[1#], Godber M Godbersen[1#], Lucas Rischka[1], Wolfgang Wadsak[2,3], Verena Pichler[2,4], Manfred Klöbl[1], Marcus Hacker[2], Rupert Lanzenberger[1], Andreas Hahn[1*]

[1] *Department of Psychiatry and Psychotherapy, Medical University of Vienna, Austria*
[2] *Department of Biomedical Imaging and Image-guided Therapy, Division of Nuclear Medicine, Medical University of Vienna, Austria*
[3] *Center for Biomarker Research in Medicine (CBmed), Graz, Austria*
[4] *Department of Pharmaceutical Sciences, Division of Pharmaceutical Chemistry, University of Vienna, Austria*

All supplementary data are formatted in tab-separated text files (.txt).

Supplementary Data 1: Data for generating Figure 2a, boxplots of control group with the following variables: m1 = PET/MRI measurement 1, m2 = PET/MRI measurement 2, easy = easy task level, hard = hard task level. Rows = subjects, columns = variables, units = score per minute.

Supplementary Data 2: Data for generating Figure 2b, boxplots of training group with the following variables: m1 = PET/MRI measurement 1, m2 = PET/MRI measurement 2, fv = final visit, easy = easy task level, hard = hard task level. Rows = subjects, columns = variables, units = score per minute.

Supplementary Data 3: Data for generating Figure 2c, training period of training group with the following variables: day 1 … day 25 = training day 1 … day 25. Rows = subjects, columns = variables, units = score per minute. NaN indicate that subjects did not train any further.

Supplementary Data 4: Data for generating Figure 3a, boxplots of control group for the insula with the following variables: m1 = PET/MRI measurement 1, m2 = PET/MRI measurement 2, rest = resting-state, easy = easy task level, hard = hard task level. Rows = subjects, columns = variables, units = MCM z-score.

Supplementary Data 5: Data for generating Figure 3a, boxplots of training group for the insula with the following variables: m1 = PET/MRI measurement 1, m2 = PET/MRI measurement 2, rest = resting-state, easy = easy task level, hard = hard task level. Rows = subjects, columns = variables, units = MCM z-score.

Supplementary Data 6: Data for generating Figure 3b, boxplots of control group for the dACC with the following variables: m1 = PET/MRI measurement 1, m2 = PET/MRI measurement 2,

rest = resting-state, easy = easy task level, hard = hard task level. Rows = subjects, columns = variables, units = MCM z-score.

Supplementary Data 7: Data for generating Figure 3b, boxplots of training group for the dACC with the following variables: m1 = PET/MRI measurement 1, m2 = PET/MRI measurement 2, rest = resting-state, easy = easy task level, hard = hard task level. Rows = subjects, columns = variables, units = MCM z-score.

Supplementary Data 8: Data for generating Figure 4a, scatterplots of training group for the association between Tetris® task performance and dACC MCM differences resting-state – hard task level with the following variables: score = Tetris® score of the hard task level during PET/MRI measurement 2, dACC rest-hard = difference in dACC MCM z-score resting-state – hard task level. Rows = subjects, columns = variables, units x-axis = MCM z-score ranked, units y-axis = score per minute ranked.

Supplementary Data 9: Data for generating Figure 4b, scatterplots of training group for the association between task performance of the training period and dACC MCM differences resting-state – hard task level with the following variables: score = Tetris® score obtained during the training period, dACC rest-hard = difference in dACC MCM z-score resting-state – hard task level. Rows = subjects, columns = variables, units x-axis = MCM z-score ranked, units y-axis = score per minute ranked.

Supplementary Data 10: Data for generating Figure 4c, scatterplots of training group for the association between mental rotation task performance and dACC MCM differences resting-state – hard task level with the following variables: Duration_by_correct = required duration divided by the number of correct answers, dACC rest-hard = difference in dACC MCM z-score resting-state – hard task level. Rows = subjects, columns = variables, units x-axis = duration/correct ranked, units y-axis = score per minute ranked.

Supplementary Data 11: Data for generating Figure 5, plots of insula with the following variables: rest = resting-state, hard = hard task level, CMRGlu = removal based on glucose metabolism, FC = removal based on functional connectivity. Rows = percentage of voxels removed from 0% to 90% in 10% steps, columns = variables, units = F-value.

Supplementary Data 12: Data for generating Figure 5, plots of dACC with the following variables: rest = resting-state, hard = hard task level, CMRGlu = removal based on glucose metabolism, FC = removal based on functional connectivity. Rows = percentage of voxels removed from 0% to 90% in 10% steps, columns = variables, units = F-value.

Supplementary Data 13: Data for generating Figure 5, plots of random removal with the following variables: rest = resting-state, hard = hard task level, dACC = dACC region, insula = insula region. Rows = percentage of voxels removed from 0% to 90% in 10% steps, columns = variables, units = F-value.
