# Supplementary material for: Learning induces coordinated neuronal plasticity of metabolic demands and functional brain networks
Source: Commun Biol. 2022 May 9;5:428. doi: 10.1038/s42003-022-03362-4 (PMC9085889; doi:10.1038/s42003-022-03362-4)
Supplement: Supplementary file 17 — Reporting Summary [file 42003_2022_3362_MOESM17_ESM.pdf]

nature portfolio

Corresponding author(s): Andreas Hahn

Last updated by author(s): Mar 15, 2022

Reporting Summary

Nature Portfolio wishes to improve the reproducibility of the work that we publish. This form provides structure for consistency and transparency in reporting. For further information on Nature Portfolio policies, see our Editorial Policies and the Editorial Policy Checklist.

Statistics

For all statistical analyses, confirm that the following items are present in the figure legend, table legend, main text, or Methods section.

n/a	Confirmed	
☐	☒	The exact sample size (*n*) for each experimental group/condition, given as a discrete number and unit of measurement
☐	☒	A statement on whether measurements were taken from distinct samples or whether the same sample was measured repeatedly
☐	☒	The statistical test(s) used AND whether they are one- or two-sided<br>*Only common tests should be described solely by name; describe more complex techniques in the Methods section.*
☒	☐	A description of all covariates tested
☐	☒	A description of any assumptions or corrections, such as tests of normality and adjustment for multiple comparisons
☐	☒	A full description of the statistical parameters including central tendency (e.g. means) or other basic estimates (e.g. regression coefficient) AND variation (e.g. standard deviation) or associated estimates of uncertainty (e.g. confidence intervals)
☐	☒	For null hypothesis testing, the test statistic (e.g. *F*, *t*, *r*) with confidence intervals, effect sizes, degrees of freedom and *P* value noted<br>*Give P values as exact values whenever suitable.*
☒	☐	For Bayesian analysis, information on the choice of priors and Markov chain Monte Carlo settings
☒	☐	For hierarchical and complex designs, identification of the appropriate level for tests and full reporting of outcomes
☐	☒	Estimates of effect sizes (e.g. Cohen's *d*, Pearson's *r*), indicating how they were calculated

Our web collection on statistics for biologists contains articles on many of the points above.

Software and code

Policy information about availability of computer code

Data collection	The video game Tetris was adapted from https://github.com/jakesgordon/javascript-tetris under the MIT license and the paradigm was implemented in electron 1.3.14. Online training was carried out with the webbrowser Chrome.<br>Cognitive tests completed outside the scanner were implemented in Matlab R2018a.
Data analysis	Preprocessing of fPET, ASL and all BOLD data was carried out in SPM12.<br>BOLD fMRI data were analyzed with SPM12.<br>fPET, ASL, BOLD functional connectivity, MCM and behavioral data were analyzed in Matlab R2018b.<br>Gray matter volume was computed with the CAT12 toolbox for SPM12.<br>White matter microstructure was analyzed with FSL 5.0.11.

For manuscripts utilizing custom algorithms or software that are central to the research but not yet described in published literature, software must be made available to editors and reviewers. We strongly encourage code deposition in a community repository (e.g. GitHub). See the Nature Portfolio guidelines for submitting code & software for further information.

Data

Policy information about availability of data

All manuscripts must include a data availability statement. This statement should provide the following information, where applicable:

- Accession codes, unique identifiers, or web links for publicly available datasets
- A description of any restrictions on data availability
- For clinical datasets or third party data, please ensure that the statement adheres to our policy

Raw data will not be publicly available due to reasons of data protection. Processed data can be obtained from the corresponding author on request with a data-sharing agreement.

Field-specific reporting

Please select the one below that is the best fit for your research. If you are not sure, read the appropriate sections before making your selection.

☒ Life sciences ☐ Behavioural & social sciences ☐ Ecological, evolutionary & environmental sciences

For a reference copy of the document with all sections, see nature.com/documents/nr-reporting-summary-flat.pdf

Life sciences study design

All studies must disclose on these points even when the disclosure is negative.

Sample size	As no longitudinal MCM studies are available, the sample size was based on previous cross-sectional work using this technique (Hahn et al. eLife 9: e52443 (2020), Riedl et al. PNAS 113: 428 (2016))
Data exclusions	53 healthy subjects were initially recruited and data from 41 healthy subjects were included in the analysis. Reasons for study drop out were voluntary discontinuation (n=6), omission to acquire fPET due to issues with arterial cannulation or radiotracer synthesis (n=3), failure of arterial blood sampling (n=2) and excessive head motion during the BOLD acquisition (n=1).<br>For n=2 subjects ASL data could not be obtained for technical reasons.
Replication	The first PET/MRI measurements of the first 22 subjects were partly analyzed twice by two different investigators to verify the reproducibility of the results at the individual level. All statistical tests at the group level were reproduced again after 4 months to confirm the initial findings by the same person originally conducting the analyses. All mentioned attempts at replication were successful.
Randomization	Participants were randomly assigned to the training or control group (dynamic balanced randomization stratified by age, sex and general intelligence).
Blinding	Data processing and analyses at the individual level were carried out blinded to group allocation.

Reporting for specific materials, systems and methods

We require information from authors about some types of materials, experimental systems and methods used in many studies. Here, indicate whether each material, system or method listed is relevant to your study. If you are not sure if a list item applies to your research, read the appropriate section before selecting a response.

Materials & experimental systems

n/a	Involved in the study	
☒	☐	Antibodies
☒	☐	Eukaryotic cell lines
☒	☐	Palaeontology and archaeology
☒	☐	Animals and other organisms
☐	☒	Human research participants
☒	☐	Clinical data
☒	☐	Dual use research of concern

Methods

n/a	Involved in the study	
☒	☐	ChIP-seq
☒	☐	Flow cytometry
☐	☒	MRI-based neuroimaging

Human research participants

Policy information about studies involving human research participants

Population characteristics	21 healthy volunteers were included in the training group (mean age ± sd = 23.0 ± 3.6 years, 11 women, mean score of general intelligence ± sd = 113.3 ± 9.5) and 20 in the control group (23.1 ± 3.1 years, 10 women, 115.1 ± 9.0).

Recruitment | Participants were recruited through poster notice boards of various universities, supermarkets, pharmacies and public advertising pillars. No selection bias was detected.

Ethics oversight | The study was approved by the ethics committee of the Medical University of Vienna (ethics number: 1479/2015).

Note that full information on the approval of the study protocol must also be provided in the manuscript.

Magnetic resonance imaging

Experimental design

Design type: resting-state, task, block design

Design specifications:
Resting-state: 1 x 6 min
Continuous task performance: 2 x 6 min for easy and hard level of difficulty
Block-design: 4 x 30 s blocks of easy, hard and control conditions each, 10 s baseline in-between, 8.17 min total

Behavioral performance measures: Tetris scoring for each completed line (score per minute) and visual control during PET/MRI acquisition by investigator. Mean ± sd or boxplots across subjects did not indicate outliers at lower performance end.

Acquisition

Imaging type(s): functional, structural, diffusion, perfusion

Field strength: 3T

Sequence & imaging parameters:
T1-weighted structural MRI: MPRAGE sequence, TE/TR = 4.21/2200 ms, TI = 900 ms, flip angle = 9°, matrix size = 240 x 256, 160 slices, voxel size = 1 x 1 x 1 mm + 0.1 mm gap, 7.72 min.
Diffusion weighted imaging: EPI sequence, TE/TR = 86/8800 ms, 64 diffusion directions with b-value = 1000 s/mm2, 6 b0-images, matrix size = 104 x 104, 70 slices, voxel size = 2 x 2 x 2 mm, 11.73 min.
ASL: 2D pseudo-continuous ASL sequence, TE/TR = 12/4060 ms, post label delay = 1800 ms, flip angle = 90°, matrix size = 64 x 64, 20 slices, voxel size = 3.44 x 3.44 x 5 mm + 1 mm gap, 3x6 min.
BOLD imaging: EPI sequence, TE/TR = 30/2000 ms, flip angle = 90°, matrix size = 80 x 80, 34 slices, voxel size = 2.5 x 2.5 x 2.5 mm + 0.825 mm gap, 3x6 min for functional connectivity and 8.17 min for neuronal activation in the block design.

Area of acquisition: Whole brain

Diffusion MRI ☒ Used ☐ Not used

Parameters: 64 directions at b = 1000 s/mm2, single shell, no cardiac gating.

Preprocessing

Preprocessing software: All BOLD data were preprocessed with SPM12. Data were corrected for slice timing effects (reference = middle slice) and head motion (quality = 1, register to mean). Spatial normalization to MNI space was carried out via the T1-weighted MRI, followed by spatial smoothing with an 8 mm Gaussian kernel.

Normalization: Spatial normalization to MNI space was carried out via the T1-weighted MRI in SPM12.

Normalization template: ICBM152 as implemented in SPM12

Noise and artifact removal: Nuisance regressors included 6 motion parameters as well as signal from white matter and cerebrospinal fluid.

Volume censoring: For functional connectivity, all frames with displacement > 0.5 mm (plus one frame back and two forward) were discarded.

Statistical modeling & inference

Model type and settings: BOLD-derived neuronal activation was assessed with a mass univariate model. Group analysis was done with a one sample t-test random effects model.

Effect(s) tested:
easy task vs. control
hard task vs. control

Specify type of analysis: ☒ Whole brain ☐ ROI-based ☐ Both

Statistic type for inference (See Eklund et al. 2016): Voxel-wise

Correction: FWE

Models & analysis

n/a	Involved in the study
☐	☒ Functional and/or effective connectivity
☒	☐ Graph analysis
☒	☐ Multivariate modeling or predictive analysis

Functional and/or effective connectivity

Voxel-wise Pearson's correlation (followed by z-tranformation) between the time courses of any given brain voxel and every voxel of the target region. The resulting connectivity pattern was used for MCM analysis.
